# Association between inter-organizational consensus on use of research evidence and stage of implementation of an evidence-based practice

**DOI:** 10.1186/1748-5908-10-S1-A20

**Published:** 2015-08-20

**Authors:** Lawrence A Palinkas, C Hendricks Brown, Lisa Saldana, Patricia Chamberlain

**Affiliations:** 1 Department of Children, Youth and Families, School of Social Work, University of Southern California, Los Angeles, CA 90089-0411, USA; 2 Department of Psychiatry and Behavioral Sciences, Feinberg School of Medicine, Northwestern University, Chicago, IL 60657, USA; 3 Oregon Social Learning Center, Eugene, OR 97401, USA

## Introduction

Two critical but poorly understood components of EBP implementation requiring collaboration across different organizations are interactions among key stakeholders and use of research evidence (URE). This paper examines the association between consensus on URE among leaders of different child-serving systems and stage of implementation of an evidence-based practice.

## Methods

Information on research evidence acquisition, evaluation and application were collected from 140 directors and senior administrators of child welfare, mental health and juvenile justice systems in 40 California and 11 Ohio counties participating in an RCT of the use of community development teams to scale up implementation of Multidimensional Treatment Foster Care over a 3 year period (2010-12). Individual data were grouped into 44 clusters, defined as 3 or more participants from 2 or more organizations in a county in a specific year. We used two separate methods for calculating consensus, cultural consensus analysis [1] of responses to the Structured Interview of Evidence Use [2], and SEM of average SIEU scale and subscale scores [3,4]. Mixed effects models were used to estimate associations between individual and group measures of URE and Stage of Implementation Completion [5] scores.

## Findings

Stage of implementation (maximum stage achieved and time to completion) was significantly associated with both measures of consensus on research evaluation (r = 0.42 p = 0.015), application (r = 0.47 p = 0.006), and engagement in overall URE (r = 0.40 p = 0.022), independent of state and year.

## Relevance to D&I

Cultural consensus measures of URE may serve two purposes for implementation science: 1) a measure of inter-organizational culture, comprised of extent of shared understandings of URE, and 2) a predictor of stage of EBP implementation that requires collaboration between multiple organizations.

## Funding

National Institute of Mental Health (R01MH076158), and the William T. Grant Foundation (#10648).
